# The role of student’s awareness of current experiences on peer and emotional problems and school refusal

**DOI:** 10.3389/fpsyg.2025.1578961

**Published:** 2025-04-07

**Authors:** Luana Sorrenti, Carmelo Francesco Meduri, Concettina Caparello, Angelo Fumia, Pina Filippello

**Affiliations:** ^1^Department of Clinical and Experimental Medicine, University of Messina, Messina, Italy; ^2^Department of Health Sciences, University Magna Graecia of Catanzaro, Catanzaro, Italy

**Keywords:** school refusal, mindfulness, peer problems, emotional problems, school wellbeing

## Abstract

**Introduction:**

Several studies have shown that awareness of present experiences/events can play a key role in promoting relational and emotional well-being as well as motivation to attend school. However, no study to date has analyzed whether a student’s awareness of current experiences (mindfulness) may contribute to peer and emotional problems and thus promote or reduce school refusal (Anxious Anticipation, Difficult Transition, Interpersonal Discomfort and School Avoidance).

**Methods:**

A cross-sectional study (362 Italian high school students) was conducted to evaluate the mediating role of peer problems and emotional problems in the association between students’ awareness of their current experiences (mindfulness) and school refusal. In order to evaluate the association between variables, was to carry out a structural equation model (SEM) with latent variables [ χ ^2^_(188)_ = 372.163, *p* = 0.000, CFI = 0.94, SRMR = 0.04, RMSEA (90%CI) = 0.052 (0.044, 0.060)].

**Results:**

Mediation analysis indicated that peer problems and emotional problems full mediate the association between mindfulness and Anxious Anticipation (peer problems: *β*_= −0.19, *p* = ≤0.001; emotional problems: *β*_= −10,*p* = ≤0.05) and Interpersonal Discomfort (peer problems: *β*_= −0.35, p = ≤0.001; emotional problems: *β*_= −0.14, *p* = ≤0.01). Moreover, peer problems fully mediate the association between mindfulness and School Avoidance (*β*_= −0.14, *p* = ≤0.01).

**Discussion:**

The study extends knowledge of the factors involved in school refusal, with application implications from a preventive point of view, relating to the components of emotional, relational and current experience awareness that may be related to motivation to attend school.

## Introduction

Scientists’ interest in mindfulness practises and experiences has increased in recent years; this is probably associated with the benefits it brings in different aspects of psychophysical well-being (Grossman et al., 2004; Veneziani and Voci, 2015). Mindfulness, a practise that has its origins in Eastern Buddhist culture, today has a particularly meaningful connotation in the psychological sphere, as it represents a state of awareness of oneself, one’s surroundings and individual experiences, paying attention to each individual moment. This moment-by-moment concentration is done without judgement and allows the individual to live their experience to the full (Kabat-Zinn, 2003). Mindfulness, described as a receptive attention and awareness of present events and experiences (Brown et al., 2007), today represents a dispositional characteristic, an individual difference that is particularly related to a person’s well-being (Baer et al., 2006). When the mind is trained to remain in the present, open, calm and alert, it is possible to better contemplate the present moment and better perceive the elements of the world around the individual (Bodhi, 1984). Through this process of awareness, the individual slowly becomes aware of who he or she really is, and trains himself or herself to see reality exactly as it is. This is the central concept of mindfulness, which emphasises the importance of living in the present, without judgement, accepting what is happening inside and outside of you. This state of presence and acceptance reduces stress and anxiety and promotes greater well-being (Kabat-Zinn, 2003). In fact, being mindful allows to face life’s challenges with greater serenity and clarity, thus improving mental and emotional health, promoting subjective well-being and reducing symptoms of anxiety, emotional and behavioural difficulties (Keng et al., 2011). Brown and Ryan (2003a) and Brown and Ryan (2003b) show that this is probably because a good, open and attentive awareness of the present moment allows the person both to reduce rumination and negative thoughts, and to develop greater emotional skills that allow them to cope better with different life situations. Such awareness and acceptance of experience without judgement is a powerful practise that can effectively counteract some forms of psycological discomfort such as anxiety, fear, anger and especially emotional, social and relational difficulties (Hayes and Feldman, 2004).

Several studies have explored at the central role that good self-awareness, awareness of one’s own experiences and feelings, plays in well-being, particularly at school (Veneziani and Voci, 2015; Bennett and Dorjee, 2016; Felver et al., 2019; Duan, 2016). In fact, most of these studies show that good self-awareness can be correlated with good school performance, low levels of anxiety and social-relational difficulties. This has important implications in the school context, as mindfulness has a positive impact on student behaviour and attendance, thereby improving both interpersonal relationships and academic success (Osteen and Smith, 2021; Nichols et al., 2021; Amitani et al., 2022). In order to respond appropriately to the demands of school each pupil should have a good understanding of his or her own strengths and difficulties (Goodman, 1997). In fact, it is important to consider students’ strengths when they are faced with particular school situations that require specific cognitive and emotional efforts, such as those used in learning processes, and relational efforts, useful in interactions with peers and teachers (Goodman, 1997). Understanding their behavioural, emotional and interpersonal profiles enables students to be more aware of their potential and the possible difficulties associated with their development (Marzocchi et al., 2002). A balanced understanding of their own psychosocial functioning, analysing both strengths and difficulties, allows children to have a more complete view of themselves, thus facilitating their own growth process. Among the strengths and difficulties that play a central role in a child’s psychological functioning are emotional problems and problems related to peer relationships. Indeed, Goodman (1997) emphasises that emotional difficulties, characterised by anxiety, somotactivity and difficulty in coping emotionally, appear to have a direct impact on the child’s daily well-being. Peer problems, such as a tendency towards isolation, difficulties in forming friendships, the possibility of becoming a victim of bullying, often result in children having difficulties in forming positive relationships and learning social skills that are crucial for their development (Sorrenti et al., 2024a; Goodman, 1997).

Previous studies have investigated how behavioural, and emotional problems in school-age children can often affect their transition from home to school (Eivers et al., 2010), resulting in problems that affect school performance (Bierman et al., 2013). Children’s social behaviour in the early years of schooling predicts later academic performance, as several studies show that students who are characterised by social difficulties in their relationships with peers (e.g., aggressiveness, poor peer interaction, etc.) require more educational support and are more likely to abandonment of school (Schwartz et al., 1998; O'Brien et al., 2007). Although most of the research on school attendance problems has been carried out mainly among early school-age children, in recent years some researchers have focused on examining this issue among adolescents (Sorrenti et al., 2024b; Gallé-Tessonneau and Gana, 2019; Gonzálvez et al., 2019). Understanding one’s own difficulties in relating to peers, in building friendships, in managing conflicts with peers, and again, difficulties in managing one’s own emotions related to anxiety and sadness, could help students cope with everyday difficulties at school (Goodman, 1999).

Consistent with the findings of some recent studies (Balakrishnan and Andi, 2019; Reid, 2005), prosocial behaviour, characterised by a good awareness of one’s peer relationship skills, is an essential skill for students to prevent peer relationship problems and problems related to school attendance. In particular, peer problems due to poor social interactions and relationships with peers can lead to school refusal (Teasley, 2004). Among the different forms of school attendance problems, school refusal (SR) is a clinical form of emotion-based absenteeism that characterises students who experience strong negative emotions when they have to go to school (Gallé-Tessonneau and Gana, 2019). SR may take various forms, including regular tardiness, frequent trips to the health clinic or school nurse, and repeated requests to call parents to leave school early (Alford, 2022; Gallé-Tessonneau and Dahéron, 2020).

Gallé-Tessonneau and Gana (2019) have identified different aspects of school refusal, such as: anxious anticipation (AA), which manifests itself in students just before they face a school situation; difficult transition (DT), characterised by difficulties in moving between home and school; interpersonal discomfort (ID), characterised by students’ discomfort with social interactions; and school avoidance (SA), characterised by avoidance of school due to anxiety, stress or other difficulties (Gallé-Tessonneau and Gana, 2019; Amitani et al., 2022). In particular, students with school refusal often show emotional difficulties and anxiety that make it difficult to get to school, to participate actively, to stay in school for the whole day and to interact with other school actors (Gallé-Tessonneau and Heyne, 2020). According to Berg (1997), school refusal is characterised by strong negative emotions that manifest themselves at the moment when a young person is in school or has to go to school. In fact, students with SR often experience reluctance or difficulty in attending school, emotional distress at the prospect of going to school, difficulties in responding to peers and teachers, and other problems that characterise relationships with peers or the demands of school (Gallé-Tessonneau and Heyne, 2020).

**Figure 1:**
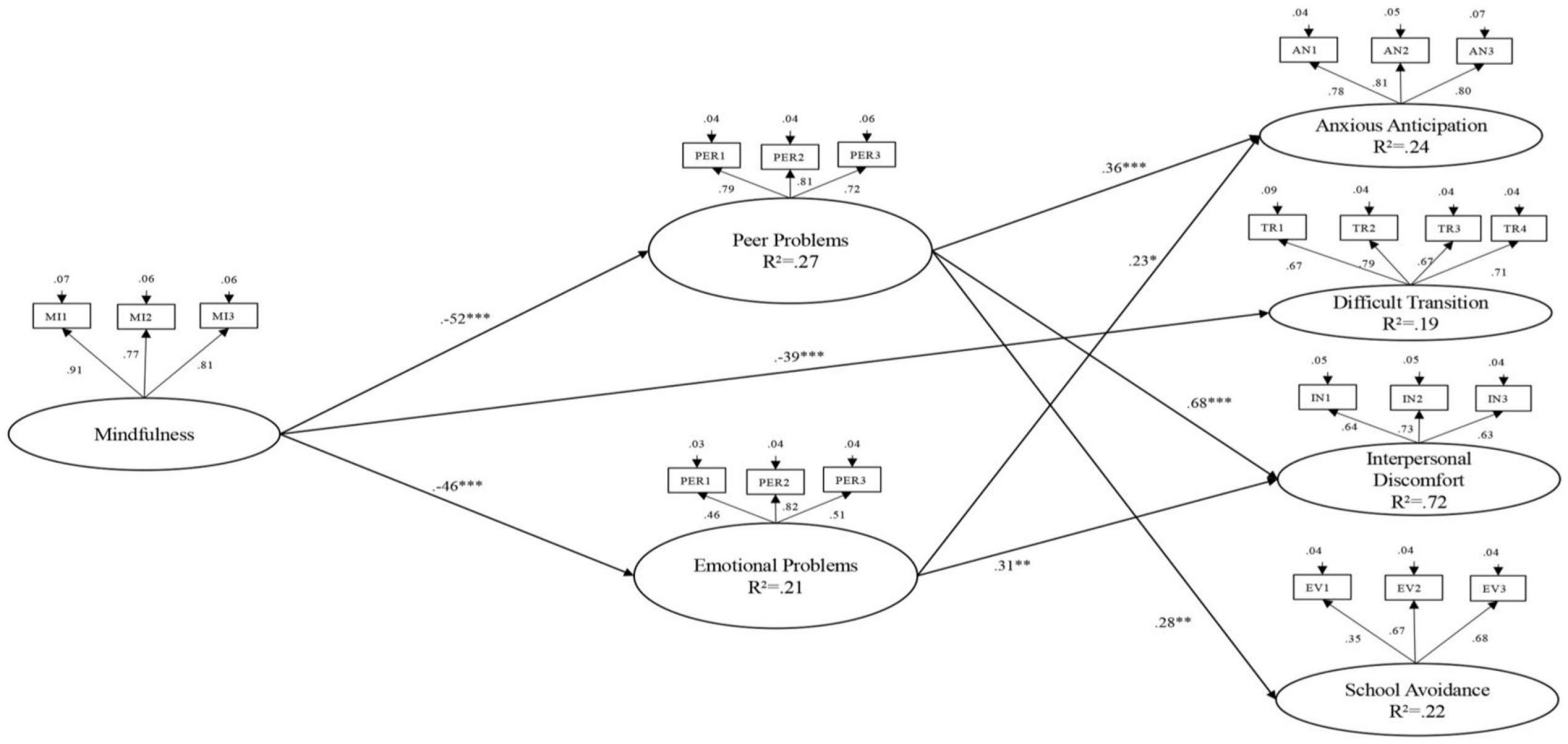
Full mediation model. ***p ≤ 0.001, *p ≤ 0.01, *p* ≤ 0.05. Coefficients shown are standardized direct path coefficients. The insignificant paths have not been inserted. Coefficients ‘Correlation: Anxious Anticipation ↔ Difficult Transition: 0.62***; Anxious Anticipation ↔ Interpersonal Discomfort: 0.45***; Anxious Anticipation ↔ School Avoidance: 0.68***; Difficult Transition ↔ School Avoidance: 0.46***; Peer Problems ↔ Emotional Problems: 0.30***.

It is important to highlight that school attendance problems are increasingly seen as problems related to social, emotional and/or behavioural difficulties in school-age children. In particular, emotional problems such as anxiety and social difficulties at school, both with peers and teachers, play a crucial role in school refusal. In fact, children who experience school refusal often show anxiety or restlessness related to stressful events at home and at school (Gonzálvez et al., 2020). In addition, these children can show poor sociability, which makes it more difficult for them to integrate into the school context (Okuyama et al., 1999). These characteristics are frequently associated with a lack of self-awareness and attention to one’s own emotions and the surrounding environment. In fact, recent studies have shown that introducing mindfulness techniques in schools could help to stabilise the mind and promote greater awareness of the present moment and one’s own strengths (Alford, 2022; Amitani et al., 2022), and this, in turn, improves emotional awareness and regulation, enhances relationships with others, and subsequently boosts academic performance. The promotion of mindfulness-based educational interventions is critical, as they not only enhance emotional awareness and regulation, but also promote improved interpersonal relationships and academic performance, thereby addressing key factors that contribute to a child’s overall psychological well-being and success in the school setting (Figure 1).

Recent researches have highlighted the importance of self-awareness, particularly understanding one’s strengths and difficulties, in promoting prosocial behaviour and positive school relationships (Lewis et al., 2021; Goodman, 1997). Emotional and peer problems, such as anxiety, social withdrawal, social relationship difficulties and peer pressure, contribute significantly to school refusal (Sorrenti et al., 2024a; Buzzai et al., 2022; Gallé-Tessonneau and Gana, 2019). Mindfulness, defined as non-judgmental awareness of the present moment, has been shown to improve well-being, social–emotional skills and consequently school performance and attendance (Nichols et al., 2021). Therefore, mindfulness can reduce mood problems and absenteeism, suggesting that mindfulness can help address school refusal related to emotional and peer difficulties (Bennett and Dorjee, 2016; Amitani et al., 2022).

Even though some studies have mostly highlighted how mindfulness-based interventions can be helpful for school attendance problems (Amitani et al., 2022; Biery, 2022; Osteen and Smith, 2021), to the best of our knowledge, no study has examined the relationship between students’ perceptions of their current experiences, emotional and peer problems, and school refusal in a single model. The literature has examined these variables separately, often highlighting the role of self-awareness in promoting adolescents’ psychological well-being (Felver et al., 2019; Hwang et al., 2018; Zoogman et al., 2015), the role of mindfulness in addressing school attendance problems (Amitani et al., 2022), and the impact of peer difficulties and emotional problems in the school context (Sorrenti et al., 2024a; Teasley, 2004; McLeod et al., 2007; Jones and Suveg, 2015). Despite these studies, literature still lacks a comprehensive model that explains the relationships between students’ awareness of their current experience, peer problems, emotional problems and SR. Therefore, the purpose of this study was to examine the mediating role of peer problems and emotional problems in the association between students’ awareness of their present experience (mindfulness) and SR (Anxious Anticipation, Difficult Transition, Interpersonal Discomfort, and School Avoidance) in a sample of adolescent students. The research on adolescent students is of interest precisely because of the increased attention in recent years to problematic issues in this age group. Indeed, it is important to consider both that adolescence is a critical period characterised by profound social, cognitive and emotional changes, and that students in this age group face greater academic pressures, social challenges and emotional fluctuations that may make them more vulnerable to emotional, social and educational problems (Lee and Song, 2017; Gallé-Tessonneau and Gana, 2019). Furthermore, tackling early school leaving in this age group is essential to prevent long-term negative outcomes such as school failure and social isolation. Based on prior research, and in accordance with Veneziani and Voci (2015) model of Mindfulness, and Gallé-Tessonneau and Gana’s (2019) model of SR, we expected that mindfulness would have a direct relationship with peer problems and emotional problems and, in turn, an indirect relationship with SR.

## Materials and methods

### Participants

The participants in this study comprised 362 Italian high school students. The mean age was 15.9 years (SD = 1.02). 152 are males (42%), 207 are females (57.2%) and 3 (0.8%) not declare their sex. Regarding the number of absences, the mean number of made absences is 20.83 (SD = 10.98) and the mean number of unexcused absences is 1.75 (SD = 7.35). Regarding students’ socioeconomic status (SES), 28% of the students belonged to a low SES (one or both parents held a lower secondary education diploma), 45.5% belonged to a medium SES (one or both parents held a high school diploma), 26.5% belonged to a high SES (one or both parents held a university degree). Furthermore, 99% of the students had Italian nationality and all participants spoke Italian. Students with intellectual disabilities or special educational needs did not participate in the research.

### Instruments

A demographic questionnaire gathered basic demographic information from participants, including their age, gender, nationality, educational level, and socioeconomic status (SES).

The Italian version of the Mindful Attention Awareness Scale (MAAS; Veneziani and Voci, 2015) was used to measure states of awareness over time. It consists of 15 items on a unidimensional scale of 7 points Likert (from 1 = almost always to 7 = almost never), all of which are aimed at investigating the child’s awareness and attention by detecting a lack of attention and awareness in specific everyday situations. In fact, as Brown and Ryan (2003a); Brown and Ryan (2003b) point out, states of unawareness are more common and easier to detect than states of awareness, so the scale is designed to detect states of unawareness in relation to states of awareness. Examples of voice: “I find it difficult to stay focused on what is happening in the present” and “I do work or tasks automatically without being aware of what I am doing.” The MAAS demonstrated acceptable reliability and construct validity in previous studies (Veneziani and Voci, 2015). In this study, the questionnaire has good internal reliabilities (*α* = 0.87).

The Italian version of the Strengths and Difficulties Questionnaire (SDQ; Marzocchi et al., 2002) is a short self-report used to identify children who need support to manage their behavioural and emotional problems. To assess peer problems (PP; e.g. “I am usually on my own; I have one good friend or more”) and emotional problems (EP; e.g. “I am often unhappy; I worry a lot”) we used two subscales of the Italian version of the test. The test provides a behavioural, relational and cognitive profile of the child. There are three versions of the test, one for parents, one for teachers and one for self-assessment. Each consists of 25 items based on a 3-point Likert scale (‘not true’, ‘partly true’, ‘absolutely true’) indicating the level of manifestation of a particular behaviour. Each subscale consisted of 5 items often used worldwide to identify children who need support to manage their behavioural and emotional problems. To assess peer problems (PP) and emotional problems (EP) we used two subscales of the Italian version of the test. In this study, the peer problems subtest has a moderate alpha value (*α* = 0.72), while the emotional problems subtest has a fairly high alpha value. (*α* = 0.79).

The School Refusal Evaluation (SCREEN; Gallé-Tessonneau and Gana, 2019) is an 18-item self-report questionnaire designed to measure different dimensions of school refusal: Anticipatory Anxiety (AA) (e.g., “I feel like I have a mental block when it comes to going to school, like I’m not able to do it”), Transition Difficulties (TD) (e.g., “I tell my parents that I do not want to go to school and that I want to stay at home”), Interpersonal Distress (ID) (e.g., “I’m afraid of what others in my class think of me”), and School Avoidance (SA) (e.g., “I’m absent more often this year than last year”). Each item is rated on a 5-point Likert scale ranging from 1 (“It does not affect me at all”) to 5 (“It affects me completely”). The SCREEN has demonstrated acceptable reliability and construct validity in previous studies (Boudouda et al., 2024; Gallé-Tessonneau and Gana, 2019; Gallé-Tessonneau and Heyne, 2020; Gallé-Tessonneau et al., 2018). In the present study, the Cronbach’s alpha value of the individual subtests is good overall. In particular, the Anticipatory Anxiety (AA) subtest has a robust alpha value (*α* = 0.83); the Transition Difficulties (TD) subtest has a high alpha value (*α* = 0.80); the Interpersonal Distress (ID) subtest has a reasonable alpha value (*α* = 0.69); and the School Avoidance (SA) subtest has a relatively high alpha value (*α* = 0.70) (Taber, 2018).

The instruments were selected for their validity and reliability in measuring the key variables of the study. In particular, the MAAS proved to be particularly useful in assessing awareness and unawareness in everyday experiences, while the SDQ, due to its concise and effective structure, allows for the reliable measurement of emotional problems and peer difficulties. Furthermore, the SCREEN was chosen for its ability to specifically analyse the four main dimensions of school refusal (Gonzálvez et al., 2021).

### Procedure

This study was performed following the recommendations of the *Ethical Code* of the *Italian Association of Psychology* (AIP) and all subjects were given written informed consent following the Declaration of Helsinki (2013). The protocol was approved by University of Alicante [UA-2023-06-29-4].

Study participants were recruited through the participation of schools in the study. After obtaining ethical approval and securing the schools’ adherence to the research project, all students in second, third, and fourth classes were informed of the project’s aims and given the opportunity to participate voluntarily and anonymously. However, only students who obtained and returned informed consent from their parents, if they were minors, or who sent their signed consent, if they were of age, were included in the research. After consent collection, the students completed the questionnaires online in a single session during school hours. The administration session, supervised by experienced staff, lasted approximately 1 hour, after which the digital questionnaires were collected and stored securely using a system of unique codes.

### Data analysis

Different software was used for the analyses. Specifically, Jamovi 2.3.28 software was used to calculate descriptive statistics and Cronbach’s alpha, while RStudio with the *lavaan* package (Rosseel, 2012) was used to perform structural equation modelling (SEM) with latent variables. By overcoming traditional univariate and multivariate methods (Iacobucci et al., 2007; Kline, 2023), the SEM approach makes it possible to reduce the likelihood of Type I errors. In addition, SEM can be used to specify latent variables that are generally assumed to be free of error (Coffman and MacCallum, 2005).

By requiring multiple indicators for each construct, SEM treats the measured constructs as latent variables. Each latent construct was measured through the specific creation of groups of items by aggregating the mean responses on a common scale of the relevant items of the questionnaire. The different groups of items for all the constructs studied allowed the structuring of the parcels that were used as main indicators (Little et al., 2007).

The parcelling procedure used in this study improves the commonality between indicators, increasing modelling efficiency and reducing random error (Bagozzi and Edwards, 1998; Coffman and MacCallum, 2005; Hau and Marsh, 2004; Kishton and Widaman, 1994; MacCallum et al., 1999; Matsunaga, 2008).

By using parcelling, model fit indices tend to be more satisfactory due to their greater psychometric and estimation advantages. In this study, calculated confidence intervals for direct and indirect effects were determined using 5,000 bootstrap replication samples. In line with the recommendations of Wu and Jia (2013), Preacher and Hayes (2008) and Shrout and Bolger (2002), a 95% bias-corrected confidence interval (CI) was used in the study. In addition, the analysis performed focused on the evaluation of the different fit indices, including the chi-square value, χ^2^/df, the comparative fit index (CFI) and the root mean square error of approximation (RMSEA) with its 90% confidence interval (CI) (Hair et al., 1998). The criteria for a good model fit were CFI values above 0.90 and RMSEA below 0.08 (Kline, 2023).

## Results

To investigate the mediating role of peer and emotional problems in the association between Mindfulness and school refusal (anxious anticipation, difficult transition, interpersonal distress and school avoidance), a structural equation model (SEM) with latent variables was used The estimation of this model produced a good fit [*χ*2 (188) = 372.163, *p* = 0.000, CFI = 0.94, SRMR = 0.04, RMSEA (90%CI) = 0.052 (0.044, 0.060)].

Results showed that PP and EP were negatively predicted by mindfulness (PP: *β* = −0.52, *p* ≤ 0.001; EP: *β* = −0.46, *p* ≤ 0.001). AA was positively predicted by PP (*β* = 0.36, *p* ≤ 0.001) and EP (*β* = 0.23, *p* ≤ 0.05). DT (difficulties in moving from home to school) was negatively predicted by mindfulness (*β* = −0.39, *p* ≤ 0.001). ID was positively predicted by PP (*β* = 0.68, *p* ≤ 0.001) and EP (*β* = 0.31, *p* ≤ 0.01). SA was positively predicted by PP (*β* = 0.28, *p* ≤ 0.001). The following significant indirect relationship of mindfulness on SR were found: from mindfulness to AA via PP (*β* = −0.19, *p* ≤ 0.001) and via EP (*β* = −0.10, *p* ≤ 0.05); from mindfulness to ID via PP (*β* = −0.35, *p* ≤ 0.001) and via EP (*β* = −0.14, *p* ≤ 0.01); from mindfulness to SA via PP (*β* = −0.14, *p* ≤ 0.01).

The results show a direct relationship between mindfulness and DT, while indirect relationships appear between mindfulness and SR (AA, ID and SA) as a result of the mediating role of PP and PE. Therefore, good awareness of the present and one’s own feelings and emotions can reduce problems with peers and emotions, which in turn can reduce SR due to AA and ID.

## Discussion

Recent studies have highlighted the importance of self-awareness, particularly in recognising one’s strengths and difficulties, in promoting pro-social behaviour and positive relationships at school (Bennett and Dorjee, 2016; Felver et al., 2019). Indeed, mindfulness, defined as non-judgmental awareness of the present moment, plays a important role in improving individual and social well-being, expecially in school setting. By increasing awareness of one’s own potential and abilities, mindfulness fosters social–emotional and behavioural skills, reduces anxiety, and thus improves school performance and attendance (Nichols et al., 2021; Amitani et al., 2022). Focusing on and becoming aware of oneself, emotions and experiences can improve interpersonal relationships and increase emotional competence (Osteen and Smith, 2021). In fact, students who are aware of their interpersonal, social and emotional competencies are more likely to feel good about themselves and their peers and perform well in school (Sorrenti et al., 2024a; Whelan-Berry and Niemiec, 2021; Bennett and Dorjee, 2016; Pickerell et al., 2023; Van de Weijer-Bergsma et al., 2014). The increased awareness of these students enables them to better manage and self-regulate the various demands of school, thus reducing problems that could affect school performance and attendance (Amitani et al., 2022).

Although several studies have analysed the relationship between mindfulness, school wellbeing and school problems such as absenteeism, no single study has analysed in a single model how awareness of oneself, one’s own experiences, emotions and feelings, can impact on different dimensions of school refusal. Therefore, this study aimed to provide preliminary support for the indirect relationship between the mindfulness and the four dimensions of school refusal through peer and emotional problems. Based on our hypotheses, we expected that mindfulness could reduce peer and emotional problems and consequently reduce the different components of school refusal. The results partly confirmed our hypotheses.

From data obtained using latent variable SEM, this study found that students with good mindfulness could have fewer peer and emotional problems, and could be less likely to experience anxiety about going to school (Anxious Anticipation), and to have difficulties with interpersonal relationships in the school setting (Interpersonal Discomfort). Conversely, students with low awareness could manifest problems with peers and could be more likely to have difficulty managing and regulating emotional and psychological distress expressed in avoidant and somatic symptoms (School Avoidance). Thus, our results thus demonstrated an association between receptive attention and awareness of current events and experiences and a reduction in emotional and peer problems, confirming previous studies (Brown et al., 2007). This is in line with the literature showing that mindfulness is positively correlated with emotional competence and stability (Veneziani and Voci, 2015) and the promotion of pro-social behaviour among peers (Balakrishnan and Andi, 2019; Reid, 2005).

In addition, our results show a direct relationship between peer and emotional problems and two dimensions of SR (Anxious Anticipation; Interpersonal Discomfort). According to Lee and Song (2017), adolescent students with difficulties in peer relationships and emotional management and regulation tend to have higher levels of anxiety related to the school experience and greater difficulties in managing school life related to interactions with peers and teachers, thus these students appear to be at greater risk of school refusal. Teasley (2004) also identifies peer problems, characterised by inadequate social interactions and relationships, as factors that characterise the avoidance behaviour typical of students with school refusal. Indeed, our research showed a direct relationship between peer problems and school avoidance. Students with emotional problems are often unable to interact effectively with others and are exposed to negative experiences (such as exclusion, peer pressure, school punishments, etc.) that increase their emotional distress and thus their disengagement from school (Teasley, 2004). When students feel safe, they feel more comfortable in the school environment, are better able to learn and promote their academic, emotional and social development. In contrast, feelings of insecurity and anxiety can often interfere with these aspects and have a negative impact on their overall wellbeing and performance (Balakrishnan and Andi, 2019).

Finally, there is a direct relationship between mindfulness and Transition Difficulties (TD); in fact, we found that students who tend to have a good level of self-awareness tend to have less difficulty transitioning from home to school. This is in line with the literature, which shows that students who perceive themselves as having a good awareness of themselves, their feelings, emotions and experiences, are able to develop specific tools that enable them to better manage the stress and anxiety factors arising from the transition from the family environment to the school environment (Boivin and Melito-Conners, 2021).

Regarding indirect effects, our study highlights the mediating role of peer and emotional problems in the relationship between mindfulness and certain dimensions of SR. In particular, we observed that good self-awareness can reduce peer and emotional problems by addressing both anxious anticipation and interpersonal discomfort. These findings may indicate that students with a good awareness of their own skills, particularly social and emotional skills, tend to have less difficulty in managing and regulating their emotions, less difficulty in relationships with peers, and this in turn may reduce school refusal problems related to anxiety and social relationships at school. In fact, according to the literature, students who have a good awareness of themselves, their environment and their individual experiences, focusing on each moment of life, tend to reflect more on themselves, experience more positive emotions, and interact well with others by building good social relationships (Schonert-Reichl et al., 2015).

Students’ perceptions of their own individual and contextual factors affecting their school life may be well related to their behaviour at school. Indeed, according to Havik and Ingul (2021), young people’s perceptions of themselves, their social relationships and their emotional abilities influence their school life, sometimes leading to negative emotions and avoidance behaviours typical of SR. The fact that peer and emotional problems play a key role in SR is also supported by Ingul et al. (2019) study, which confirms this relationship. Indeed, the way in which young people regulate their emotions (i.e., monitor, evaluate and address emotional reactions) and manage their relationships with peers is of interest because both emotional difficulties and social problems with peers are essential features of SR. In fact, while self-awareness is important in recognising one’s own feelings and behaviour, it is not necessarily accompanied by the ability to manage and resolve emotional or relational problems. Developing concrete tools to deal with emotional difficulties or to improve social skills can help to manage school life, success and attendance (King and Bernstein, 2001). Finally, another important indirect effect is the mediating role of peer problems in the association between mindfulness and school avoidance. Our findings are consistent with studies by Dube and Orpinas (2009) who show that students who manifest school avoidance are often characterised by behavioural difficulties such as conduct problems, hyperactivity and problems with peers. Probably, self-awareness, by adopting a non-judgmental attitude towards oneself, tends to reduce socio-relational difficulties with peers and provides students with the tools they need to cope with the demands of school, which in turn reduces school avoidance (Table 1).

**Table 1:** Path estimates, standard errors (SE), and 95% confidence intervals (CIs).

Indirect Effect Path	*β*	SE	95% CI (Lower BC)	95% CI (Upper BC)	*p*-value
Indirect effect via peer problems					
Mindfulness → Anxious Anticipation	−0.19	0.02	−0.15	−0.04	≤ 0.001
Mindfulness → Interpersonal Discomfort	−0.35	0.03	−0.24	−0.10	≤ 0.001
Mindfulness → School Avoidance	−0.14	0.01	−0.07	−0.01	≤ 0.01
Indirect effect via emotional problems					
Mindfulness → Anxious Anticipation	−0.10	0.02	−0.11	−0.01	≤ 0.05
Mindfulness → Interpersonal Discomfort	−0.14	0.02	−0.12	−0.02	≤ 0.01

## Limitations and future directions

This study has several limitations that need to be considered when interpreting the results. One of the main limitations is that the survey has a cross-sectional design, which means that data were collected at a single point in time. This design limits our ability to draw conclusions about causality: whether awareness directly affects peer problems and emotional problems, which in turn affect school rejection, or whether other factors may be at play. To establish causal relationships and better understand the long-term effects of awareness on school refusal, future research should use experimental and longitudinal designs. Longitudinal experimental studies of consciousness, such as self-awareness training programmes or guided meditation practises, could help to understand how increasing self-awareness leads directly to the well-being of adolescents by reducing school refusal. In addition, longitudinal studies could follow students over time, allowing for a more thorough and comprehensive investigation. In fact, such studies would make it possible to observe how self-awareness and related psychological, social and environmental factors associated with school refusal develop at different stages of school life. Another limitation of the study concerns the use of a convenience sample consisting of high school students from southern Italy, which may limit its representativeness compared to the general population. Furthermore, the sample does not exhibit a homogeneous distribution with respect to both educational levels and gender differences. For this reason, future studies should include students from various educational levels and countries to conduct a cross-cultural analysis. Additionally, it would be beneficial to consider more homogeneous samples or to conduct targeted analyses to explore gender differences in greater detail. Cultural and demographic differences may also play a crucial role in understanding the phenomenon of school refusal, as factors such as the school climate, peer and teacher relationships, family expectations, and available school resources vary across different cultural contexts, shaping students’ school experiences. The use of self-report questionnaires is another limitation, as participants may respond in ways that are expected or socially acceptable, rather than giving honest answers. To overcome this limitation, future research should include additional tools such as direct observation. In addition, interviews or focus groups with students, teachers and parents could provide richer and more qualitative insights into the complex dynamics between awareness and school refusal. Furthermore, it is important to recognise the possible presence of potential confounding variables, such as individual differences in the experience of awareness. Future studies should include more detailed measures to assess and control such differences in order to examine more precisely their impact on the process under investigation.

Despite these limitations, our study contributes to the growing interest in school awareness and refusal and highlights how awareness of one’s problems with peers and emotions plays a key role in this association.

First, to the best of our knowledge, given the current interest of researchers in mindfulness, this is the first study to examine the role of awareness, particularly of social problems with peers and emotional problems, in coping with school refusal. In addition, the study focuses on a sample of adolescents, a particular stage of development often characterized by problems with school attendance (Kearney et al., 2020). Adolescence is a crucial period for the growth and development of social and emotional skills. It is a phase in which young people may be more vulnerable to difficulties such as anxiety, stress management and a sense of belonging in the school context (Feiss et al., 2019). Given the benefits of mindfulness interventions for individual and social well-being in the school context, it would be appropriate to promote programs in schools aimed at enhancing social, emotional and cognitive skills that promote greater self-awareness. While previous research have highlighted the usefulness of mindfulness interventions based on promoting greater attention to the present in increasing psychological well-being (Felver et al., 2019; Hwang et al., 2018; Zoogman et al., 2015), our research has focused on examining the role of awareness of one’s strengths and weaknesses in the relationship between mindfulness and school refusal. Recent studies have confirmed that mindfulness significantly contributes to reducing stress and school problems, thereby improving the psychological well-being of adolescents (Amitani et al., 2022; Felver et al., 2019; Hwang et al., 2018; Zoogman et al., 2015). In particular, mindfulness interventions realised in presence, such as the Learning to BREATHE programme (Felver et al., 2019) and implemented in an online environment (Biery, 2022), have been found to be particularly effective in improving students’ well-being, performance and school behaviour. In addition, mindfulness yoga interventions have recently been developed to support children in coping with anxiety related to school refusal (Amitani et al., 2022). Considering these findings, our study illustrates how the implementation of such mindfulness interventions, by designing activities aimed at increasing awareness of one’s strengths, in this case by paying attention to one’s emotional and relational problems with peers, could contribute to improving students’ school attendance and well-being. Indeed, encouraging students to become aware of their strengths by paying attention to their relational, social and emotional skills is crucial to promoting their well-being and improving their academic performance (Whelan-Berry and Niemiec, 2021; Bennett and Dorjee, 2016). In line with Bennet and Dorjee’s research (2016), having a good awareness of current events, different experiences, and even one’s own feelings and emotions promotes well-being and reduces school-related problems, particularly mood, social and emotional problems such as absenteeism. This heightened awareness enables individuals to develop better management and self-regulation skills, allowing them to respond adaptively to the different demands of school, and thus reducing negative symptoms. Moreover, mindfulness practises, by fostering greater awareness of one’s abilities and the learning process, can facilitate the development of effective academic strategies, supporting students in cultivating advanced academic competencies while promoting increased engagement and attendance at school (Osteen and Smith, 2021; Buzzai et al., 2021).

## Data Availability

The raw data supporting the conclusions of this article will be made available by the authors, without undue reservation.
